# 6-Gingerol, a Major Constituent of *Zingiber officinale* Rhizoma, Exerts Anticonvulsant Activity in the Pentylenetetrazole-Induced Seizure Model in Larval Zebrafish

**DOI:** 10.3390/ijms22147745

**Published:** 2021-07-20

**Authors:** Kinga Gawel, Wirginia Kukula-Koch, Nancy Saana Banono, Dorota Nieoczym, Katarzyna M. Targowska-Duda, Lidia Czernicka, Jolanta Parada-Turska, Camila V. Esguerra

**Affiliations:** 1 Chemical Neuroscience Group, Centre for Molecular Medicine Norway, Faculty of Medicine, University of Oslo, Gaustadalléen 21, 0349 Oslo, Norway; n.s.banono@ncmm.uio.no (N.S.B.); c.v.esguerra@ncmm.uio.no (C.V.E.); 2 Department of Experimental and Clinical Pharmacology, Medical University of Lublin, Jaczewskiego Str. 8b, 20-090 Lublin, Poland; 3 Chair and Department of Pharmacognosy, Medical University of Lublin, Chodzki Str. 1, 20-093 Lublin, Poland; virginia.kukula@gmail.com; 4 Department of Animal Physiology and Pharmacology, Institute of Biology and Biochemistry, Faculty of Biology and Biotechnology, Marie Curie-Skłodowska University, Akademicka Str. 19, 20-033 Lublin, Poland; dnieoczym@gmail.com; 5 Department of Biopharmacy, Medical University of Lublin, Chodźki Str. 4a, 20-093 Lublin, Poland; katarzyna.duda@umlub.pl; 6 Chair and Department of Food and Nutrition, Medical University of Lublin, Chodzki Str. 4a, 20-093 Lublin, Poland; lidia.czernicka@umlub.pl; 7 Department of Rheumatology and Connective Tissue Diseases, Medical University of Lublin, 20-090 Lublin, Poland; jolanta.turska@umlub.pl

**Keywords:** *Zingiber officinale*, isolation, 6-gingerol, zebrafish, pentylenetetrazole, seizures, anticonvulsant activity, EEG, neurotransmitter profiling, molecular docking

## Abstract

*Zingiber officinale* is one of the most frequently used medicinal herbs in Asia. Using rodent seizure models, it was previously shown that *Zingiber officinale* hydroethanolic extract exerts antiseizure activity, but the active constituents responsible for this effect have not been determined. In this paper, we demonstrated that *Zingiber officinale* methanolic extract exerts anticonvulsant activity in the pentylenetetrazole (PTZ)-induced hyperlocomotion assay in larval zebrafish. Next, we isolated 6-gingerol (6-GIN)—a major constituent of *Zingiber officinale rhizoma*. We observed that 6-GIN exerted potent dose-dependent anticonvulsant activity in the PTZ-induced hyperlocomotion seizure assay in zebrafish, which was confirmed electroencephalographically. To obtain further insight into the molecular mechanisms of 6-GIN antiseizure activity, we assessed the concentration of two neurotransmitters in zebrafish, i.e., inhibitory γ-aminobutyric acid (GABA) and excitatory glutamic acid (GLU), and their ratio after exposure to acute PTZ dose. Here, 6-GIN decreased GLU level and reduced the GLU/GABA ratio in PTZ-treated fish compared with only PTZ-bathed fish. This activity was associated with the decrease in *grin2b*, but not *gabra1a*, *grin1a*, *gria1a*, *gria2a*, and *gria3b* expression in PTZ-treated fish. Molecular docking to the human NR2B-containing N-methyl-D-aspartate (NMDA) receptor suggests that 6-GIN might act as an inhibitor and interact with the amino terminal domain, the glutamate-binding site, as well as within the ion channel of the NR2B-containing NMDA receptor. In summary, our study reveals, for the first time, the anticonvulsant activity of 6-GIN. We suggest that this effect might at least be partially mediated by restoring the balance between GABA and GLU in the epileptic brain; however, more studies are needed to prove our hypothesis.

## 1. Introduction

Epilepsy, a chronic neurological disorder that affects approximately 1% of the population worldwide, is characterized by recurrent, atypical brain activity, which is clinically manifested through various symptoms, such as loss of consciousness and unpredictable convulsions [1]. Although our understanding of the pathophysiology of epilepsy has improved within the last 10 years [2,3,4], there is still an ongoing need for identifying new drug leads with better safety profiles and higher efficacy than currently available antiseizure drugs (ASDs) [5]. This is especially the case for 30% of patients who do not respond to such drugs [6]. To fulfill this gap in clinical practice, new animal models suitable for high-throughput screening of new drug leads are desired. Among them, zebrafish (*Danio rerio*) is now widely accepted by the scientific community as a valuable model for this purpose (for review, see [7,8]). What makes this model organism attractive for epilepsy research, among others, is its rapid development, high fecundity, and small size at the larval stage allowing for concomitant screening of several drugs within the same time frame and high genomic homology to mammals [9].

In larval zebrafish, seizures are induced by bathing the animal in a solution of one of the following: pentylenetetrazole (PTZ) [10], allylglycine [11], or ethyl ketopentenoate [12]. However, the most commonly used among them is PTZ, which induces behavioral and local field potential (LFP) changes in larval zebrafish, an equivalent of tonic-clonic seizures in humans [10]. Here, the behavioral component of seizures is manifested as hyperlocomotion. In the LFP recordings, a high number of epileptiform-like discharges are observed [10,13]. Compounds with antiseizure activity diminish PTZ-induced hyperlocomotion and decrease the number of LFP based epileptiform-discharges [7,14]. Using this model, the antiseizure activity of various plant-derived constituents have been identified, e.g., oliganthin H from *Garcinia oligantha* [15], palmatine from *Berberis sibirica* [13], indirubin from *Indigofera arrecta* [16], or bisabolene sesquiterpenoids from *Curcuma longa* [17].

The ginger rhizomes (*Zingiber officinale* Roscoe, Zingiberaceae) belong to the most frequently used medicinal herbs. The plant, which originates from Asia, has been widely used in recipes of traditional medicine around the world due to its antiemetic, antibacterial, antiviral, anti-inflammatory, bile production enhancing, body weight reducing, antioxidant, and anticancer properties [18]. The aforementioned pharmacological effects of the plant are mainly related to the presence of terpenes and phenolic acid derivatives in its oleoresin. The latter components, called gingerols and shogaols, which are responsible for the pungent taste of the rhizome, were recently proven to cross the blood–brain barrier [19] and exhibit central activity that includes the monoamine oxidase A (MAO-A) inhibition [20], migraine relief [21], dendritic cell activity amelioration [22], and neuroprotective action [23]. Previously, *Zingiber officinale* hydroethanolic extract was shown to exert antiseizure activity in the acute intravenous PTZ test and PTZ-kindling model in mice, but active constituents responsible for this effect have not been determined [24,25]. More recently, using the PTZ-induced hyperlocomotion assay in larval zebrafish, Brillatz et al. [26] indicated that the hexane extract of *Zingiber purpureum* possesses anticonvulsant properties, with two active phenylbutenoids, i.e., *trans*- and *cis*-banglene, being responsible for this effect.

In the current study, we first assessed the effect of the methanolic extract from ginger rhizomes in the PTZ-induced hyperlocomotion assay in larval zebrafish. Next, we evaluated the influence of 6-gingerol (6-GIN), a major constituent isolated from *Zingiber officinale* rhizoma methanolic extract, on the PTZ-induced hyperlocomotion in zebrafish larvae. Here, we indicated, for the first time, the potent and dose-dependent antiseizure activity of 6-GIN. The inhibitory activity on seizure paroxysmal behavior in zebrafish larvae was confirmed by LFP recordings in larval brains. To gain further insight into the anticonvulsant activity of 6-GIN, we measured the concentration of two neurotransmitters, i.e., inhibitory γ-aminobutyric acid (GABA) and excitatory glutamic acid (GLU), which are known to be dysregulated in epileptic patients [27,28,29]. Additionally, we measured the concentration of serotonin (5-HT) [27,28] and dopamine (DA) [29,30]. Next, using quantitative real-time polymerase chain reaction (qRT-PCR), we analyzed the expression of a few genes encoding neurotransmitter receptors (namely *gabra1a*, *grin1a*, *grin2b, gria1a, gria2a*, and *gria3b*) to quantify possible changes in expression. Lastly, we performed in silico study to explain the possible inhibitory activity of 6-GIN and propose the binding sites of 6-GIN at the NR2B-containing N-methyl-D-aspartate (NMDA) receptor.

## 2. Results

### 2.1. The Effect of Zingiber officinale Rhizome Methanolic Extract in the PTZ-Induced Hyperlocomotion Assay in Larval Zebrafish

We first determined the maximum tolerated concentration (MTC) of *Zingiber officinale* rhizome methanolic extract. For this, 4-day-old zebrafish larvae were incubated for 24 h in different concentrations of *Zingiber officinale* rhizome methanolic extract. Next, each larva was individually investigated under the microscope for signs of toxicity/malformations. The following traits were scored: loss of posture, morphological malformations, swim bladder appearance, and changes in heart rate or rhythm. Additionally, the escape response upon a light touch of the tail and hypoactivity were assessed. The dose of 60 µg/mL extract, which did not affect any of the above-mentioned scores, was considered the MTC for further investigation.

**Figure 1:**
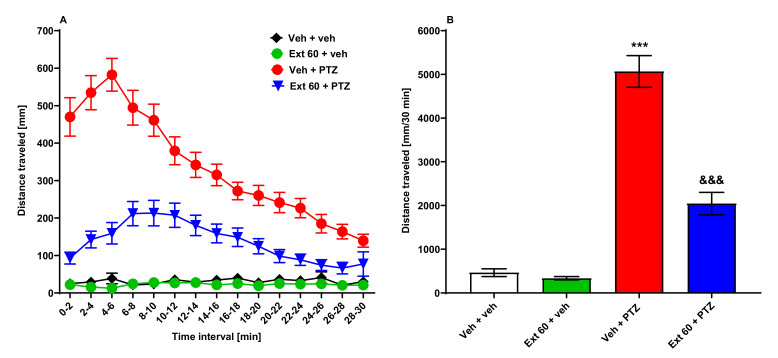
The influence of *Zingiber officinale* rhizoma methanolic extract on seizure-like behavior in the PTZ-induced hyperlocomotion assay in zebrafish. After 24 h incubation in *Zingiber officinale* rhizoma methanolic extract (60 µg/mL), 7-day-old zebrafish larvae were exposed to an acute dose of PTZ (20 mM). Larval behavior was assessed 5 min after PTZ application. Results of the experiment are depicted as: (**A**) distance covered by larvae in 2 min long time bins, and (**B**) total distance covered by larvae during 30 min of the assay. Data were analyzed using one-way or two-way ANOVA with repeated measures followed by Tukey’s or Bonferroni’s *post hoc* test, respectively. Data are depicted as a mean ± standard error of the mean (SEM). Veh + veh (*n* = 31), Ext 60 + veh (*n* = 24), Veh + PTZ (*n* = 48), Ext 60 + PTZ (*n* = 48). *** *p* < 0.001 vs. Veh + veh; ^&&&^ *p* < 0.001 vs. Veh + PTZ. Ext 60—*Zingiber officinale* methanolic extract (60 µg/mL), PTZ—pentylenetetrazole, Veh—vehicle. *n*—refers to the total number of larvae.

After 24 h incubation with *Zingiber officinale* extract (60 µg/mL) or vehicle (Veh), 7-days post-fertilization (dpf) zebrafish were exposed to an acute dose of PTZ (20 mM). Two-way ANOVA with repeated measures yielded significant differences among the tested groups of animals (group: F(3, 2205) = 441.8, *p* < 0.001; time point: F(14, 2205) = 9.72, *p* < 0.001; group × time point interaction: F(42, 2205) = 7.15, *p* < 0.001; *n* = 24–48/group; Figure 1A). Additionally, one-way ANOVA of total distance traveled revealed the differences among groups (F(3, 147) = 31.41, *p* < 0.001; *n* = 24–48/group; Figure 1B). Here, Tukey’s *post hoc* test showed that PTZ significantly increased locomotor activity of larvae compared with the animals treated with Veh (*p* < 0.001). Pretreatment with *Zingiber officinale* rhizome methanolic extract for 24 h reduced PTZ-induced locomotor activity by 50% (*p* < 0.001). The ginger extract itself did not influence larval activity, as compared to the Veh-treated group (*p* > 0.05, Figure 1A,B).

### 2.2. The Isolation of 6-GIN from Ginger Extract

The identification of 6-GIN was performed based on data recorded by the HPLC-ESI-QTOF-MS/MS spectrometer, which included the retention time and the fragmentation pattern of this metabolite. In addition, the mass spectra database (Metlin) and the scientific literature were used to confirm compound identity. The 6-GIN ion observed in the positive ionization mode spectra was present as a sodium adduct [M-Na]^+^ and had the *m/z* value of 317 Da. The MS/MS spectra recorded in the collision energy of 5 and 10 V showed the presence of one major product ion with the *m*/*z* of 170 Da, which was in accordance with previous studies [31]. This fragment appeared as a detachment product of the substituted phenolic ring.

**Figure 2:**
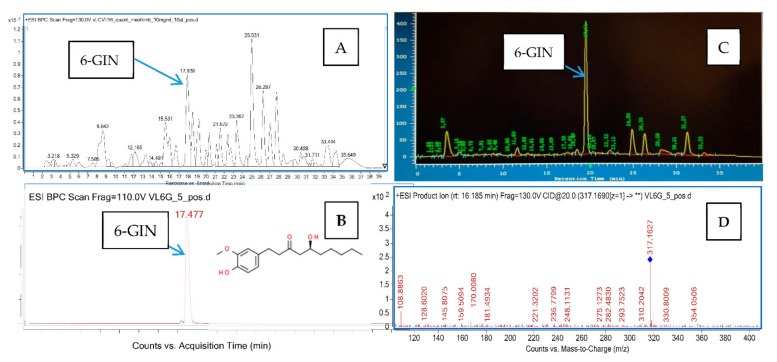
The chromatograms and fragmentation spectra obtained for the total extract and the isolated 6-GIN. (**A**) the total ion chromatogram of the methanolic extract from *Zingiber officinale* rhizome recorded in the positive ionization mode; (**B**) the total ion chromatogram of the isolated 6-GIN recorded in the positive ionization mode; (**C**) the chromatogram from the semipreparative HPLC, recorded at 290 nm; and (**D**) the MS/MS fragmentation spectrum of 6-GIN.

The isolation of 6-GIN from the methanolic extract of ginger rhizomes was performed on a semi-preparative chromatographic column. The composed chromatographic method provided relatively short runs (40 min) (Figure 2). 6-GIN was eluted in the 19^th^ minute and, in the wavelength of 290 nm, it was the major peak present in the extract. The final purity of the compound was 98.3% (Figure 2).

### 2.3. The Effect of 6-GIN in the PTZ-Induced Hyperlocomotion Assay in Larval Zebrafish

**Figure 3:**
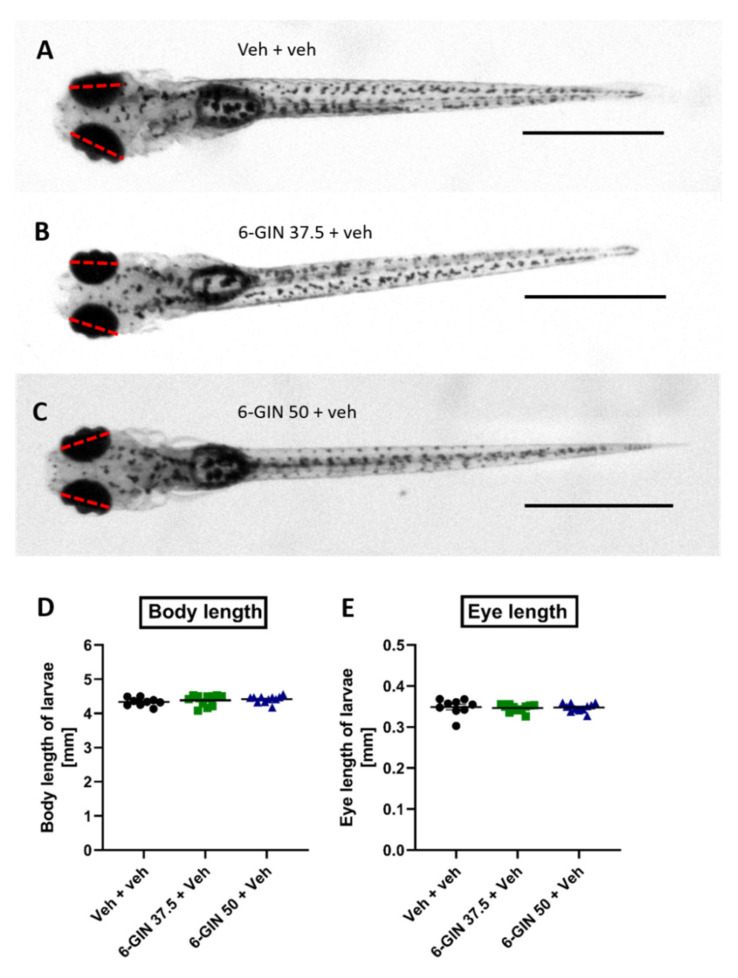
Larvae treated with 6-GIN are morphologically indistinguishable from their Veh-treated counterparts. (**A**) Larva treated with Veh for 24 h (*n* = 9). (**B**) Larva treated with 37.5 µM 6-GIN for 24 h (*n* = 11). (**C**) Larva treated with 50 µM 6-GIN for 24 h (*n* = 11). (**D**) Comparison of body length of Veh- and 6-GIN-treated larvae (**E**) Comparison of eye length of Veh- and 6-GIN-treated larvae. Red dash lines represent how eye length was measured, scale bar = 1 mm. 6-GIN—6-gingerol, Veh—vehicle.

Similarly, as for *Zingiber officinale* rhizome extract, we first determined the MTC value for 6-GIN. The MTC assay was conducted as described above. The dose of 50 µM 6-GIN did not change any scores apart from hypoactivity (presumably due to sedation). However, to exclude that this hypoactivity might be related to developmental delay, we measured the larval body length and eye size in 5 dpf fish after 24 h incubation with 50 µM. There was no difference between treated and non-treated larvae in terms of body length and eye size (*p* > 0.05, Figure 3A,B,D,E). Thus, this concentration was excluded from further investigation to avoid false-positive results in the PTZ-induced seizure assay. The highest concentration of 6-GIN, which was devoid of this side effect (i.e., did not cause hypoactivity and developmental delay), was 37.5 µM and was considered as the MTC for all subsequent experiments (Figure 3A,C,D,E).

**Figure 4:**
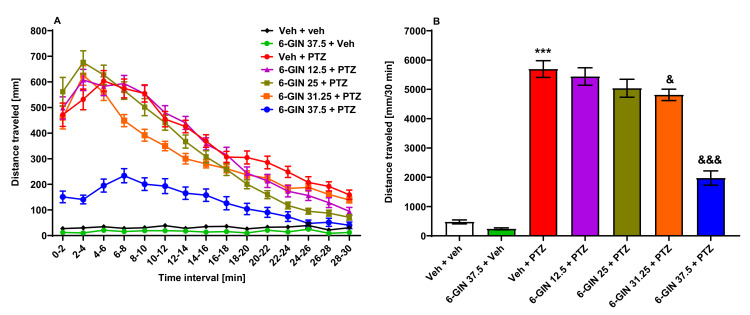
The influence of 6-GIN on the seizure-like behavior in the PTZ-induced zebrafish hyperlocomotion assay. After 24 h incubation in different doses of 6-GIN (12.5, 25, 31.25, or 37.5 µM), 7-day-old zebrafish larvae were exposed to PTZ (20 mM). Larval behavior was assessed 5 min after PTZ administration. Results of the assay are depicted as: (**A**) distance covered by larvae in 2 min long time bins, and (**B**) total distance covered by larvae during 30 min of the assay. Data were analyzed using one-way or two-way ANOVA with repeated measures followed by Tukey’s or Bonferroni’s *post hoc* test, respectively. Data are depicted as a mean ± SEM. Veh + veh (*n* = 48), 6-GIN 37.5 + Veh (*n* = 48), Veh + PTZ (*n* = 64), 6-GIN 12.5 + PTZ (*n* = 47), 6-GIN 25 + PTZ (*n* = 56), 6-GIN 31.25 + PTZ (*n* = 48), 6-GIN 37.5 + PTZ (*n* = 37). *** *p* < 0.001 vs. Veh + veh; ^&&&^ *p* < 0.001, ^&^ *p* < 0.05 vs. Veh + PTZ. 6-GIN—6-gingerol, PTZ—pentylenetetrazole, Veh—vehicle. *n*—refers to the total number of larvae.

Next, 6-day-old larval zebrafish were incubated for 24 h with different doses of 6-GIN (12.5, 25, 31.25 or 37.5 µM) or Veh and subsequently exposed to an acute dose of PTZ (20 mM). Statistical analysis conducted with two-way ANOVA with repeated measures showed differences among tested groups of zebrafish larvae (group: F(6, 341) = 99.86, *p* < 0.001; time point: F(14, 1378) = 214.60, *p* < 0.001; group × time point interaction: F(84, 4774) = 23.05, *p* < 0.001; *n* = 37–64/group; Figure 4A). Moreover, one-way ANOVA of total distance traveled revealed the differences among groups (F(6, 341) = 99.86, *p* < 0.001; *n* = 37–64/group; Figure 4B). Here, Tukey’s *post hoc* test showed that 6-GIN in a dose of 31.25 and 37.5 µM decreased PTZ-induced hyperlocomotor activity (*p* < 0.05 and *p* < 0.001, respectively). The two lower doses of 6-GIN (i.e., 12.5 and 25 µM) did not affect PTZ-induced hyperlocomotor activity (*p* > 0.05) as compared to only PTZ-treated animals. The 6-GIN (37.5 µM) itself did not influence the larval activity (*p* > 0.05, Figure 4A,B).

Based on the data described above for 6-GIN, the concentration reducing PTZ-induced hyperlocomotion by 50% (ED_50_) was calculated. 6-GIN inhibited PTZ-induced hyperlocomotion behavior with ED_50_ value equal to 36.0 (34.2–37.9) µM, r = 0.89.

### 2.4. The Influence of 6-GIN on PTZ-Induced LFP Discharges in Zebrafish

**Figure 5:**
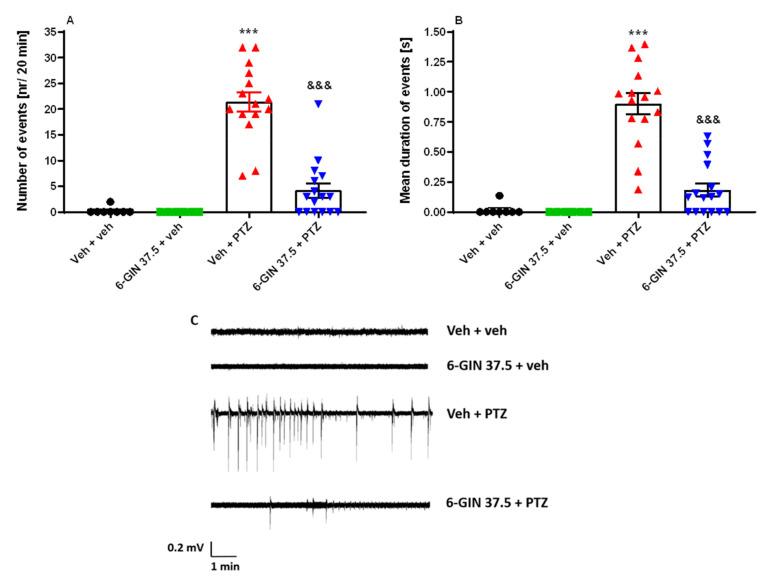
LFP events detected from the optic tectum of 7-day-old zebrafish larvae pre-exposed for 24 h to 6-GIN (37.5 µM). Subsequently, each larva was incubated with 20 mM PTZ or Veh for 5 min, before being mounted in agarose for LFP recordings. Data are shown as: (**A**) the number of events (nr/20 min), and (**B**) the mean duration of events (s/20 min). Data were analyzed using one-way ANOVA followed by Tukey’s *post hoc* test. Data are depicted as mean ± SEM. Veh + veh (*n* = 8), 6-GIN 37.5 + Veh (*n* = 6), Veh + PTZ (*n* = 14), 6-GIN 37.5 + PTZ (*n* = 16). *** *p* < 0.001 vs. Veh + veh; ^&&&^ *p* < 0.001 vs. Veh + PTZ. (**C**) Representative traces of recordings. 6-GIN—6-gingerol, PTZ—pentylenetetrazole, Veh—vehicle. *n*—refers to the total number of larvae.

To confirm our behavioral assay observations, we performed LFP recordings to assess seizure modulation by 6-GIN in larval brains [8]. Here, one-way ANOVA of LFP events detected from the optic tectum of 7 dpf larvae yielded statistically significant differences between tested groups of animals regarding the number (F(3, 41)  =  42.53, *p* < 0.001; *n* = 6–16/group; Figure 5A) and mean duration of events (F(3, 41)  =  38.20, *p* < 0.001; *n* = 6–16/group; Figure 5B). 6-GIN (37.5 µM) significantly reduced the number (*p* < 0.001) and mean duration of epileptiform-like discharges (*p* < 0.001) in comparison to the PTZ-treated group. 6-GIN (37.5 µM) did not exert any effect on the brain activity of Veh-treated larvae (*p* > 0.05) (Figure 5A,B; for representative recordings, see Figure 5C).

### 2.5. The Influence of 6-GIN on the Concentration of GABA, GLU and Their Ratio, as Well as DA and 5-HT in PTZ-Treated Zebrafish Larvae

**Figure 6:**
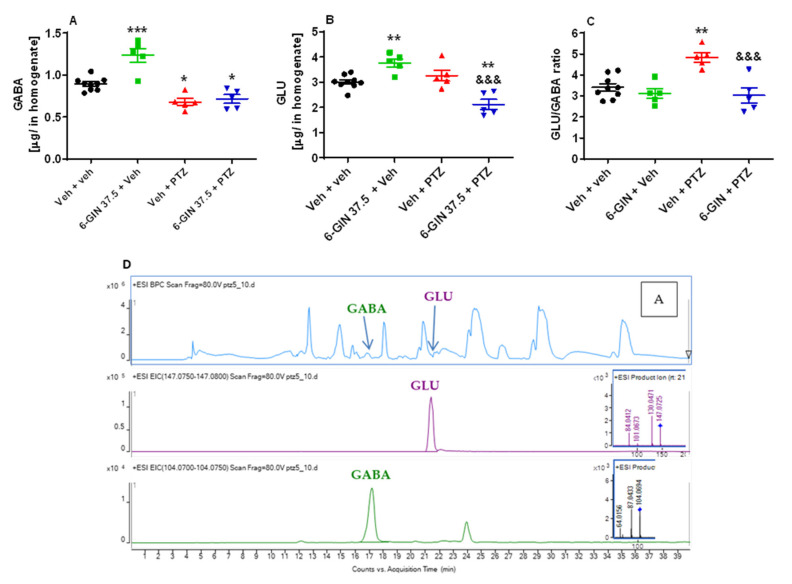
Determination of (**A**) GABA, (**B**) GLU, (**C**) GLU/GABA ratio, and (**D**) the total ion chromatogram (**A**) of the water: acetonitrile extract from zebrafish larvae with extracted ion chromatograms and MS/MS spectra of GLU and GABA in 7-day-old zebrafish larvae by HPLC-MS. After a 24 h incubation in 6-GIN (37.5 µM), zebrafish larvae were exposed to an acute dose of PTZ (20 mM) for 90 min. Next, whole zebrafish larvae were collected in a pool of *n* = 100/sample. Data were analyzed using one-way ANOVA followed by Tukey’s *post hoc* test. Data are depicted as mean ± SEM. Veh + veh (*n* = 9), 6-GIN 37.5 + Veh (*n* = 5), Veh + PTZ (*n* = 5), 6-GIN 37.5 + PTZ (*n* = 5). *** *p* < 0.001, ** *p* < 0.01, * *p* < 0.05 vs. Veh + veh; ^&&&^ *p* < 0.001 vs. Veh + PTZ. 6-GIN—6-gingerol, PTZ—pentylenetetrazole, Veh—vehicle. *n*—refers to the total number of samples.

To determine the influence of 6-GIN on the concentration of GABA, GLU and their ratio, as well as DA and 5-HT in PTZ-treated zebrafish, 6-day old larvae were incubated for 24 h with 37.5 µM 6-GIN or Veh. Next, larvae were exposed to an acute dose of PTZ (20 mM) for 90 min. One-way ANOVA yielded statistically significant differences between tested groups of animals (GABA: F(3, 20)  =  23.78, *p* < 0.001; *n* = 5–9/group; Figure 6A; GLU: F(3, 20)  =  16.52, *p* < 0.001; *n* = 5–9/group; Figure 6B; GLU/GABA ratio: F(3, 20)  =  10.52, *p* < 0.001; *n* = 5–9/group; Figure 6C; 5-HT: F(3, 20) (3, 5.57, *p* < 0.01; *n* = 5–9/group; Supplementary Figure S1A; DA: F(3, 20) (3, 25.69, *p* < 0.01; *n* = 5–9/group; Supplementary Figure S1B). Tukey’s *post hoc* analysis showed that the concentration of GABA was decreased (*p* < 0.05), whereas the level of GLU was not affected in the PTZ-treated group (*p* < 0.05), compared with the Veh-treated group. Pre-exposure to 37.5 µM 6-GIN in PTZ-treated larvae decreased GABA (*p* < 0.05) level compare with Veh- but not with PTZ-exposed zebrafish. In the case of GLU, pre-exposure to 37.5 µM 6-GIN in PTZ-treated larvae decreased its content in comparison with both Veh- (*p* < 0.05) and PTZ-exposed (*p* < 0.01) zebrafish. 6-GIN (37.5 µM) itself increased levels of GABA (*p* < 0.001) and GLU (*p* < 0.01) as compared with only Veh-treated fish. Nevertheless, when GLU to GABA ratio was analyzed, Tukey’s *post hoc* analysis revealed that this ratio was increased only in PTZ-treated fish (*p* < 0.01). Pre-exposure to 37.5 µM 6-GIN in PTZ-treated larvae reversed this tendency, as compared with PTZ-exposed fish (*p* < 0.001).

Concerning 5-HT and DA, Tukey’s *post hoc* analysis revealed that 6-GIN and PTZ given singly did not affect their levels (*p* > 0.05) as compared with the control group. Interestingly, the level of 5-HT was decreased (*p* < 0.01) whereas the level of DA was increased (*p* < 0.001) in 6-GIN + PTZ-treated fish, as compared with only the PTZ-treated group (Supplementary Figure S1A,B and Supplementary Figure S2). This indicates that 6-GIN + PTZ exhibit some interaction, which deserves further investigation.

### 2.6. The Influence of 6-GIN on gabra1a, grin1a, grin2b, gria1a, gria2a, and gria3b Expression in PTZ-Treated Zebrafish Larvae

To gain further insight into the anticonvulsant activity of 6-GIN, we analyzed the expression of a few genes encoding receptors for GABA (*gabra1a*) and GLU (*grin1a, grin2b*, i.e., NMDA receptors; and *gria1a*, *gria2a*, and *gria3b*, i.e., AMPA receptors) using qRT-PCR. *gabra1a* is a zebrafish orthologue of the mammalian gene encoding the α1 subunit of the GABA receptor. This subunit is critical for the formation of the ion channel and plays a pivotal role in GABA_A_ receptor functions—together with α2, α3, and α5 subunits, conferring them sensitivity to benzodiazepines [32]. Moreover, its involvement in seizure disorders both in experimental animals, including rodents as well as zebrafish [7,33], and patients [34,35] was previously unequivocally displayed. Transcripts of *gabra1a* were observed in the developing zebrafish beginning at 18 h post-fertilization (hpf) [33]. Glutamatergic NMDA and AMPA-type receptors also play significant roles in the generation of epileptic disorders [36,37,38]. Hunt et al. [36] reported that the expression of *grin1a* and *grin2b* subunits of NMDA receptor in zebrafish larvae is low both at 2 and 3 dpf and is significantly increased during the following days of development. Nevertheless, a more detailed analysis revealed *grin1a* expression in the forebrain, hindbrain, midbrain, spinal cord, and presumptive neural retina of the embryonic zebrafish at 24 hpf [39,40]. *grin1a* expression in the brain of the adult zebrafish is very high [41]. Evaluation of *grin2b* mRNA expression in the whole developing zebrafish was visible at 96 hpf [40,42]. *gria1a, gria2a* and *gria3b* belong to the group of eight paralogues genes that encode subunits of glutamatergic AMPA type receptors. Transcripts of *gria1a, gria2a*, and *gria3b* were found in the developing brain structures of zebrafish larvae as early as 24 hpf, and their expression increased in the following hours of development [43].

**Figure 7:**
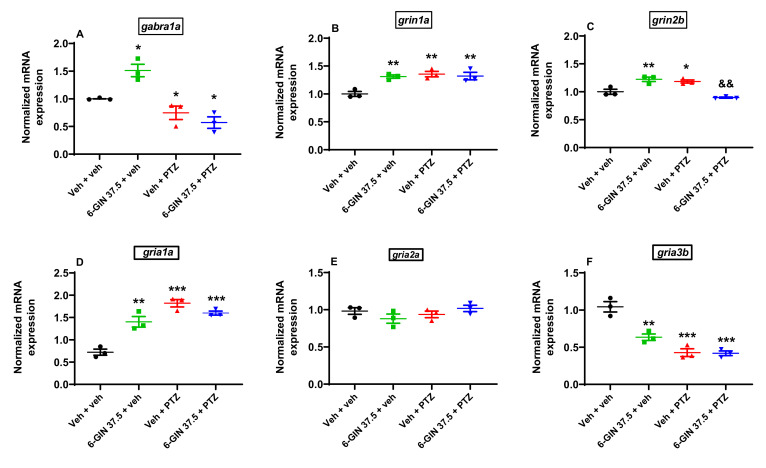
The effect of 6-GIN on (**A**) *gabra1a*, (**B**) *grin1a*, (**C**) *grin2b,* (**D**) *gria1a,* (**E**) *gria2a*, and (**F**) *gria3b* mRNA expression in PTZ-treated zebrafish larvae. After a 24 h incubation in 6-GIN (37.5 µM), zebrafish larvae were exposed to PTZ (20 mM) for 90 min. Next, zebrafish larvae were collected in a pool of *n* = 10/sample. mRNA levels were normalized against *gapdh.* Data were analyzed using one-way ANOVA with Tukey’s *post hoc* test. Data are depicted as a mean ± SEM (*n* = 3/group). *** *p* < 0.01, ** *p* < 0.01, * *p* < 0.05 vs. Veh + veh; ^&&^ *p* < 0.01 vs. Veh + PTZ. 6-GIN—6-gingerol, PTZ—pentylenetetrazole, Veh—vehicle. *n*—refers to the total number of samples.

In our study, after 24 h incubation with 37.5 µM 6-GIN or Veh, 7-day-old larvae were exposed to an acute dose of PTZ (20 mM) for 90 min. qRT-PCR expression analysis with one-way ANOVA revealed statistically significant differences in the expression levels of *gabra1a* (F(3, 11) = 17.53, *p* < 0.001; *n* = 3/group; Figure 7A); *grin1a* (F(3, 11) = 11.31, *p* < 0.01; *n* = 3/group; Figure 7B); *grin2b* (F(3, 11) = 21.16, *p* < 0.001; *n* = 3/group; Figure 7C); *gria1a* (F(3, 11) = 32.87, *p* < 0.001; *n* = 3/group; Figure 7D); and *gria3b* (F(3, 11) = 32.93, *p* < 0.001; *n* = 3/group; Figure 7F); but not *gria2a* (F(3, 11) = 1.49, *p* > 0.05; *n* = 3/group; Figure 7E). Tukey’s *post hoc* test indicated that PTZ induced substantial decrease in the expression of *gabra1a* (*p* < 0.05) and *gria3b* (*p* < 0.001) but an increase in the expression of *grin1a* (*p* < 0.001), *grin2b* (*p* < 0.05), and *gria1a* (*p* < 0.001), compared with Veh-treated larvae. Pre-exposure to 37.5 µM 6-GIN significantly downregulated *grin2b* (*p* < 0.01) but not *gabra1a*, *grin1a, gria1a*, *and gria3b* (both *p* > 0.05) expression in PTZ-treated larvae, compared with only PTZ-treated fish. Interestingly, in the absence of PTZ, *gabra1a* (*p* < 0.05), *grin1a, grin2b*, *and griaa1a* (all *p* < 0.01) in 6-GIN (37.5 µM) + Veh-treated group was found to be upregulated compared with Veh-treated group. In the absence of PTZ, *gria3b* was downregulated (*p* < 0.01). There was no difference between the tested groups of animals with regard to *gria2a* expression.

### 2.7. Molecular Docking of 6-GIN to NR2B-Containing NMDA Receptor

**Figure 8:**
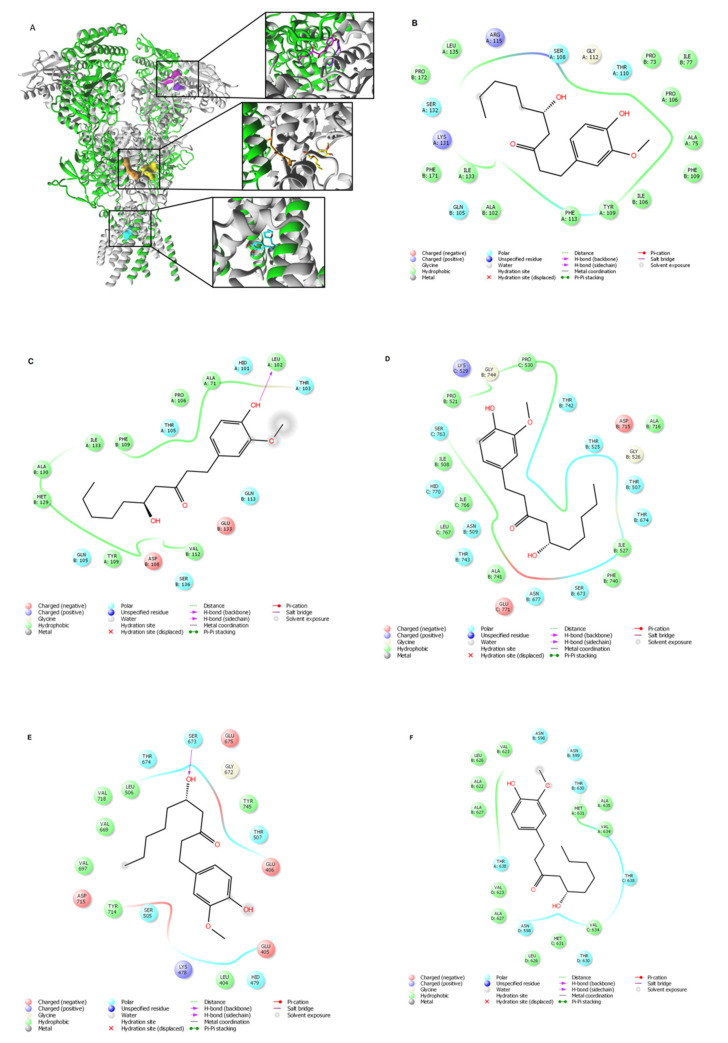
Molecular docking of 6-GIN to the GluN1/GluN2B NMDA receptor. (**A**) 6-GIN binding sites (surface model) are located at the ATD (magenta and purple), the LBD (yellow and orange) and the TMD (cyan) of the GluN1/GluN2B NMDA receptor. More specifically, molecular docking results suggested two different orientations of 6-GIN at ATD, a high-affinity site (magenta) located at the GluN1/GluN2B interface and a low-affinity site (purple) within the GluN2B subunit. In addition, 6-GIN may interact with the glutamate binding site (at the LBD). Orientation 1 presents a higher affinity site (orange) at the GluN1/GluN2B LBD interface, and orientation 2 presents a lower affinity pose (yellow) located within the LBD of the GluN2B subunit. Finally, the 6-GIN interacts within the ion channel (at the TMD) of the GluN1/GluN2B NMDA receptor. (**B**,**C**) 2D views of 6-GIN interacting with the high-affinity site and low-affinity site at the ATD, respectively. (**D**,**E**) 2D views of 6-GIN interacting with the higher-affinity as well as lower-affinity sites at the LBD, respectively. (**F**) 2D view of the 6-GIN interactions within the ion channel pore of the GluN1/GluN2B NMDA receptor. The GluN1 subunits are shown in green, and the GluN2B subunits are depicted in grey. The residues involved in 6-GIN binding are presented in panels (**B**–**F**) (2D views) and included in Table 1. Hydrogen bonds are marked with pink arrows, residues involved in hydrophobic interactions are shown in green, polar in blue, and charged residues involved in binding are shown in red.

To determine the binding site locations and structural components for 6-GIN at the NR2B-containing NMDA receptor, we performed a molecular docking analysis of 6-GIN on the crystal structure of the human GluN1/GluN2B NMDA receptor. In the case of the selected model, all subunits possess a modular domain architecture, with amino-terminal domains (ATDs), ligand-binding domains (LBDs) that are localized on the extracellular side of the membrane, transmembrane domain (TMD) spanning the membrane and defining the ion channel pore, and an intracellular carboxy-terminal domain (CTD). 6-GIN was docked to ATDs, LBDs, and TMD of the GluN1/GluN2B NMDA receptor. Molecular docking results suggested two different orientations of 6-GIN at ATD, a high-affinity site located at the GluN1/GluN2B interface and a low-affinity site within the GluN2B subunit (Figure 8A–C). The residues involved in 6-GIN binding at ATD, LBD, and TMD are presented in Table 1. In addition, 6-GIN may interact with the glutamate binding site (at the LBD). Orientation 1 presents a higher affinity site at the GluN1/GluN2B LBD interface (Figure 8A,D; Table 1) and orientation 2, which represents a lower affinity pose, is located within the LBD of the GluN2B subunit (Figure 8A,E; Table 1). Finally, 6-GIN interacts within the ion channel pore (at TMD) of the GluN1/GluN2B NMDA receptor (Figure 8A,F; Table 1). Of note, all amino acid residues involved in 6-GIN binding to presented binding sites are very conserved (identical) and are present in both human and zebrafish NR2B-containing NMDA receptor sequence.

Finally, multiple alignments of GluN2B subunit sequences from humans and zebrafish were generated. It confirmed that the residues involved in 6-GIN binding are identical in both human and zebrafish sequences of the GluN2B subunit.

## 3. Discussion

6-GIN was identified in the methanolic extract from *Zingiber officinale* as a major component. Since ginger rhizomes constitute a very rich source of metabolites of different chemical character, from different types of terpenes to phenolic acids [44], the preparative HPLC technique was selected for the recovery of this cinnamic acid derivative [45]. Previous attempts to isolate this compound by counter-current chromatography based on the already published protocols [46,47] led to the recovery of 6-GIN together with terpenes that were decreasing the purity of the isolate. After all, the proposed technique provided high purity 6-GIN (98.3%) from the rhizome extract.

Since ginger extract constituents, including 6-GIN, have the ability to pass the blood–brain barrier [19], we postulated that they might modulate central nervous system functions. Moreover, their beneficial effects were previously reported in Parkinson’s disease, Alzheimer’s disease [48,49,50,51], depression [52,53], stress [54], and anxiety [55,56] in animal models. The anticonvulsant effect of ginger extract in the intravenous PTZ seizure test in mice has also been reported [24,25]. However, neither the influence of individual ingredients of the ginger extracts, mainly gingerols and shogaols, nor the mechanism of anticonvulsant activity of ginger extracts/constituents has been previously investigated.

In the present study, we demonstrated the anticonvulsant effect of *Zingiber officinale rhizoma* methanolic extract in the zebrafish larvae PTZ seizure assay. Interestingly, the study carried out in the PTZ-treated zebrafish larvae exposed to *Zingiber officinale* rhizome methanolic extract by Brillatz and colleagues [26] did not reveal this effect. It is worth noting that the compositional analysis of *Zingiber* *officinale* reported by these authors did not reveal the presence of 6-GIN in the extract. Instead, they noted a significant reduction of the PTZ-induced hyperlocomotion in the zebrafish larvae incubated with the hexane extract from the rhizome of *Zingiber purpureum* in which *trans*- and *cis*-banglene were identified as the main active compounds. As the composition of *Zingiber officinale* differs significantly from the herein described extract (a detailed composition was reported by the authors in the previously published manuscript [44]) it is difficult at this point to compare the anticonvulsant action of our reported *Zingiber officinale* extract with the previous data of Brillatz and co-workers [26]. At this point, it is important to underline that the activity of plant extracts analyzed in in vivo models may differ depending on the climatic conditions, access to water and nutrients, and so on, as shown in previous studies on different plant species [57,58]. Gingerols are the components of ginger extracts that are present in the fresh plant material. Longer storage, elevated temperature, age of the plant, or location may be responsible for the transformation of gingerols to shogaols [59], which is a result of the dehydration of the molecule. These factors might have played an important role in the plant species studied by Brillatz and colleagues [26].

Numerous studies carried out using the PTZ-induced seizure test in mice found anticonvulsant properties of *Zingiber officinale* extracts. Both sub-chronic (daily by 1 week) as well as acute (2 and 24 h before the seizure test) administration of hydroethanolic extract of *Zingiber officinale* significantly increased thresholds for the myoclonic twitches, generalized clonic and tonic seizures induced by intravenous PTZ infusion in mice [24,25]. Moreover, anticonvulsant properties of chronically administered ginger extract (for 2 weeks) in the intravenous PTZ seizure threshold test were also noted in the streptozocin-induced diabetic mice [60]. Our findings indicate that 6-GIN might be one of the active compounds of *Zingiber officinale* extract, which is responsible for its properties, including anticonvulsant activity. Incubation of zebrafish larvae with 6-GIN attenuated the PTZ-induced seizure-like behavior, which was manifested as a significant reduction of hyperlocomotion as well as a decrease in the number of LFP events detected in the recordings obtained from the optic tectum of the larvae.

Although the PTZ-induced seizure tests are the most common models used in the preclinical screening of compounds with anticonvulsant properties, the precise mechanism of PTZ action is not known. It is widely considered that seizures result mainly from antagonism by PTZ on the benzodiazepine site and blockade of the chloride ion channel in the GABA receptor complex, which attenuates the inhibitory GABAergic neurotransmission [61,62]. Previous studies revealed that PTZ-induced seizures, both in rodents as well as in zebrafish larvae, are inhibited mostly by compounds that enhance GABAergic neurotransmission [10,63,64]. The present findings and results of the studies in mouse PTZ tests indicate that the anticonvulsant action of ginger extracts and 6-GIN might be connected to their influence on GABA neurotransmission.

Concentrations of GABA and GLU—two key neurotransmitters in the central nervous system underlying the expression of seizure discharges—were determined to elucidate the possible mechanism of anticonvulsant action of 6-GIN in our study. The applied chromatographic conditions provided efficient separation of the low molecular mass metabolites present in zebrafish larvae. The separation was achieved on a ReproSil-Pur 120 Si chromatographic column (Dr. Maisch, Ammerbuch, Germany). The application of this particular column, which is characterized by the presence of a fully porous bidentate phase, resulted in the good separation of GABA and GLU. These two neurotransmitters were eluted together at the beginning of the chromatograms developed with standard RP-18 phases. The proposed column, which provides better stability and higher resistance to acidic and basic mobile phases was found efficient enough to separate small high polarity molecules similar to HILIC-type columns [65] in a gradient that starts from an organic phase and has an increasing volume of the aqueous phase on the column. The applied conditions enabled the quantification of the two selected neuroactive molecules whose presence is crucial in the analysis of antiseizure effects of new drug candidates. The conditions used made it possible to perform the quantitative analysis on zebrafish larvae, which is quite difficult considering the size of larvae and the measured concentration levels of the neurotransmitters. To date, this type of quantitative research has been carried out almost exclusively on the brains of larger animals, such as mice and rats, in which the concentration of neurotransmitters reached higher levels [66,67].

As we expected, exposure of zebrafish larvae to PTZ significantly reduced GABA concentrations and, although there was no change in the level of GLU, the GLU/GABA ratio was significantly higher. These results indicate that PTZ affects mainly the GABAergic system and in this way dysregulates the balance between excitatory glutamatergic and inhibitory GABAergic neurotransmission, which, in consequence, leads to seizure activity. Twenty-four-hour incubation of zebrafish larvae with 6-GIN increased both GABA and GLU concentration but did not affect the balance expressed as GLU/GABA ratio. Interestingly, in the PTZ-treated group, 6-GIN did not influence GABA concentration (vs. the control PTZ-treated group) but significantly reduced GLU level (both in comparison with the Veh + PTZ-treated group as well as with the Veh + veh group). Although we expected that the anticonvulsant action of 6-GIN results mainly from its influence on GABAergic neurotransmission, the above-mentioned results suggest that it might rather result from the attenuation of glutamatergic neurotransmission, which counters the PTZ-induced imbalance between excitatory and inhibitory neurotransmitters in the zebrafish larvae. There are only limited data on the influence of ginger extracts and their constituents on neurotransmitter concentrations in brain structures, but they are consistent with our results. Studies conducted by Hussein et al. [68] revealed that the neuroprotective effect of ginger against monosodium glutamate neurotoxicity in rats resulted, among others, from the significant reduction of the cerebral cortex GLU concentration. Ginger supplementation upregulated GABA level in the hippocampus and cortex of the senile female rats. Moreover, there was also noted a significant increase in the hippocampal GLU level [69].

To assess the influence of 6-GIN more accurately on the GABAergic and glutamatergic systems, we evaluated the mRNA expression of GABA, NMDA, and AMPA receptor subunits using the qRT-PCR method. Changes in these receptors’ composition might alter their pharmacokinetic properties, affect these two neurotransmission systems, and make an impact on seizure activity. Unfortunately, changes in mRNA expression were not confirmed by studies of the respective protein expression. Western blotting could not be performed due to an insufficient amount of tissue collected from zebrafish and the unavailability of the respective zebrafish-specific antibodies.

In our study, PTZ treatment significantly downregulated *gabra1a* mRNA expression in the zebrafish larvae, which suggests that its convulsant effect might be, among others, mediated by changes in the subunit composition of GABA_A_ receptors, exactly by the decrease in α1 subunit contribution, which might weaken inhibitory GABAergic signaling. Although 6-GIN significantly increased *gabra1a* mRNA expression in comparison to the Veh + veh group, it did not change its expression in the PTZ-treated zebrafish larvae, which again indicates that the protective effect of 6-GIN may not be connected to the influence on GABAergic neurotransmission.

Evaluation of mRNA expression levels for two glutamate NMDA receptor subunits, i.e., *grin1a* and *grin2b*, gave us results that again support the influence of 6-GIN on the glutamatergic system. Both PTZ and 6-GIN alone upregulated *grin1a* and *grin2b* expression in the zebrafish. 6-GIN did not influence *grin1a* expression in zebrafish larvae exposed to PTZ, but significantly reduced the level of *grin2b* transcripts. This change might weaken NMDA receptor activity and glutamatergic neurotransmission and, as a consequence, induce an anticonvulsant effect in the PTZ-treated zebrafish larvae. To explain the possible mechanism of 6-GIN inhibitory activity toward the GluN2B-containing NMDA receptor, molecular docking was used. Our data suggest that 6-GIN may interact with the ATD and glutamate-binding site as well as within the ion channel.

Since the involvement of AMPA receptors in the process of icto- and epileptogenesis has been proven (for reviews, see [38,70]), we would expect that all 3 genes (*gria1a, gria2a*, and *gria3b*) might be upregulated in the PTZ-treated fish. Nevertheless, only *gria1a*, but not *gria2a* (no effect) and *gria3b* (downregulation), was upregulated. Interestingly, *gria1a* and *gria3b* expression at the mRNA level in the 6-GIN + PTZ-treated group did not differ significantly from both 6-GIN- and PTZ-treated groups. It seems, at least under our experimental conditions, that 6-GIN does not affect the seizure-related phenotype in PTZ-treated larvae through modulation of AMPA receptors. Thus, the influence of 6-GIN on AMPA receptor subunits expression should be studied more precisely to establish interactions with glutamatergic neurotransmission.

In conclusion, the previously reported protective effect of ginger extracts in the models of seizures and epilepsy might be related, among others, to 6-GIN content. Our results show anticonvulsant properties of 6-GIN for the first time. We suggest that this effect might be, at least partially, mediated by restoring the balance between GABA and GLU in the epileptic brain. Regarding the mRNA study, it is important to note that alterations observed after 90 min of PTZ treatment on mRNA levels may not necessarily influence protein levels. Thus, in the future, Western blot analysis in, e.g., a zebrafish model of Dravet syndrome (*scn1lab* deficiency [71]), will be done to prove our hypothesis. We also suggest that 6-GIN inhibits the NR2B-containing NMDA receptor through interaction with ATD and the glutamate-binding site, as well as within the ion channel. Further evaluation of the influence of 6-GIN on other neurotransmitter systems and pathways that have been suggested to be involved in epilepsy disorders [27], as well as usage of other seizure/epilepsy models is still warranted. Based on obtained data, it cannot be unambiguously concluded, whether the effects seen are directly related to the 6-GIN treatment or indirectly through the enhancement of seizure threshold and/or reduction of seizure severity. Following the promising data presented in this study, there is the need to continue the search for further therapeutic properties of 6-GIN as well as other constituents of *Zingiber officinale* extracts.

## 4. Materials and Methods

### 4.1. Drugs and Reagents

PTZ was purchased from Sanofi Aventis (Paris, France). The solvents used for the extraction were obtained from Avantor Performance Materials (Gliwice, Poland). The preparative HPLC isolation of 6-GIN was performed using chromatography grade acetonitrile (Merck, Darmstadt, Germany) and water obtained from Merck Millipore system (Darmstadt, Germany). All solvents used for the HPLC-MS analysis (water, acetonitrile, and formic acid of HPLC-MS purity grade) were purchased from Merck (Darmstadt, Germany). The obtained extract was filtered with nylon syringe filters (0.22 µm) produced by Merck (Darmstadt, Germany). The standards of GABA and GLU were purchased from Sigma Aldrich (St. Louis, MO, USA).

### 4.2. Extraction

Fresh rhizomes of *Zingiber officinale* were purchased from a local market in Lublin, Poland. The plant material was finely sliced and dried in the oven at a temperature of 35 °C for 5 consecutive days. The dried material was pulverized and stored in closed amber glass containers prior to extraction. Methanolic extracts from powdered ginger rhizomes were prepared in 66 mL steel vessels using ca. 15 g of plant material by accelerated solvent extractor (ASE 100, Dionex, Sunnyvale, CA, USA) in the following conditions: static time: 10 min, temperature: 80 °C, pressure: ca. 110 bar, the number of extraction cycles: 3, purge volume: 80%, and purge time: 40 s.

The collected extracts were joined and evaporated under pressure on a rotary evaporator at the temperature of 40 °C.

### 4.3. Chromatographic Analysis of the Extract by HPLC-ESI-QTOF-MS/MS

The composition of the extract was determined by an HPLC chromatograph (1200 Series) coupled with mass spectrometer: HPLC-ESI-QTOF-MS/MS (6500 Series) by Agilent Technologies (Santa Clara, CA, USA). The chromatograph was equipped with a degasser (G1322A), a binary pump (G1312C), an autosampler (G1329B), a PDA detector (G1315D), and a QTOF mass spectrometer (G6530B). The qualitative analysis was performed in both ionization modes, according to the previously published protocol with modifications [44]. The chromatographic column Zorbax Eclipse Plus (dimensions: 150 mm, 2.1 mm, d_p_ = 3.5 µm) from Agilent Technologies (Santa Clara, CA, USA) provided an effective separation of the extracts’ constituents in the following gradient of acetonitrile with 0.1% formic acid (B) in 0.1% formic acid in water: 0 min 30% B; 10 min 45% B; 15 min 65% B; 25 min 80% B; 30 min 90% B; and 31 min 30% B. The flow rate was set at 0.2 mL/min, the injection volume at 10 µL, and the run length at 40 min. The mass chromatograms were recorded on a freshly calibrated instrument with the addition of internal standards mixture by Agilent Technologies (Santa Clara, CA, USA) under the following conditions: capillary voltage 3500 V; gas and sheath gas temperatures: 350 and 400 °C; gas and sheath gas flows of 12 L/min, respectively; nebulizer pressure 35 psig; skimmer voltage 65 V; fragmentor voltage 110 V; collision energies of 10 and 20 V; and nozzle voltage 1000 V and within the range of 100–1500 *m*/*z*. The Mass Hunter Workstation Software (version B.08.00) by Agilent Technologies (Santa Clara, CA, USA) was used to recording and handle the spectrometric data.

### 4.4. Isolation of 6-GIN from the Total Extract by Semi-Preparative HPLC Chromatography

An HPLC chromatograph LaChrom D-7000 by Merck Hitachi, composed of degasser, a quaternary pump (L-7150), a UV-VIS detector (L-7420), and a sample collector (SF-3120), was used for the purification of 6-GIN from the methanolic extract from *Zingiber officinale* rhizomes. The separation was performed on a semi-preparative C18-AR-II column Cosmosil (250 × 10 mm, 5 µm) (Nacalai Tesque, Kyoto, Japan).

The following gradient of acetonitrile (B) in water was elaborated to isolate 6-GIN from the extract at high purity: 0 min 37% B, 10 min 50% B, 14 min 65% B, 20 min 72% B, 30 min 80% B, and 32 min 37% B. The flow rate was set at 3 mL/min, the analysis time at 40 min, and the detection wavelength at 290 nm. The isolation was performed by injecting 50 µL of the crude extract at a concentration of 100 mg/mL. The HPLC System Manager (version 4.0) (Merck, Darmstadt, Germany and Hitachi, San Jose, CA, USA) was used for the collection of chromatograms and data handling.

### 4.5. Zebrafish Maintenance and Breeding

Wildtype adult zebrafish of the AB strain, purchased from the Zebrafish International Resource Center (Eugene, OR, USA), were raised and kept under standard housing conditions (at 28.5 °C on a 14-h light/10-h dark cycle). Embryos resulting from natural spawning were maintained in E3 embryo water (1.5 mM HEPES, pH 7.6, 17.4 mM NaCl, 0.21 mM KCl, 0.12 mM MgSO_4_ and 0.18 mM Ca(NO_3_)_2_), at 28.5 °C and on a 14-h/10-h light/dark cycle. Larvae up to 7 days post-fertilization (dpf) were used for all assays. All animal experiments were approved by the Norwegian Food Safety Authority experimental animal administration’s supervisory and application system (“Forsøksdyrforvatningentilsyns-og søknadssystem”, FOTS-ID 15469 and 23935). Furthermore, compliance with the ARRIVE and the National Institute of Health Guidelines for the Care and Use of Laboratory Animals, the European Community Council Directive of November 2010 for Care and Use of Laboratory Animals (Directive 2010/63/EU) guidelines were applied to all experiments.

### 4.6. Evaluation of Maximum Tolerated Concentration Doses of Tested Compounds

4-dpf zebrafish larvae were incubated with *Zingiber officinale* rhizome methanolic extract or 6-GIN solutions for 24 h. After, each larva was individually investigated under a stereomicroscope (Zeiss Stemi2000C, Zeiss, Jena, Germany) for signs of toxicity. The following traits were scored: loss of posture, morphological malformations, swim bladder appearance, and changes in heartbeat. Additionally, escape response upon a touch of the tail and hypoactivity were assessed. Only doses of compounds that did not affect any of the above-mentioned scores were considered to be MTC doses for the purpose of our experiments.

### 4.7. Morphological Phenotyping

5-dpf larvae were imaged after 24 h incubation in 6-GIN (37.5 or 50 µM) using a Leica M205 FA microscope, and measurements of body and eye lengths were performed using ImageJ. Body length was represented as the distance from the tip of the head to the end of the caudal fin. Eye length was measured as the widest distance from the dorsal to the ventral extremity of the eye. The lengths of both eyes from individual larva were measured and the average value used.

### 4.8. Locomotor Activity Measurement in Larval Zebrafish

Locomotor activity experiments were conducted as described in our previous papers [13,14]. Briefly, 6 dpf larvae were placed individually in the wells of a 48-well plate filled with 200 µL of Veh, *Zingiber officinale* rhizome methanolic extract (dose: 60 µg/mL) or 6-GIN (doses: 12.5, 25, 31.25, or 37.5 µM) solutions. Following this, larvae were incubated for 24 h at 28.5 °C. Subsequently, Veh or PTZ (final concentration 20 mM) was added to each well. After a 5-min delay (habituation), larval activity was tracked (ZebraBox, Viewpoint, Lyon, France) for 30 min with 2-min time bins. The distance covered by each larva in millimeters (mm) was assessed. The measurements were two or three times replicated and the data were pooled together.

### 4.9. LFP Recordings in Larval Zebrafish

LFP measurements were performed from zebrafish larval optic tecta at 7 dpf, as described originally by Afrikanova et al. [10]. Here, each larva was mounted on a glass slide in a thin layer of 2% low-melting-point agarose. Then, the glass electrode (resistance 1–5 MΩ) filled with artificial cerebrospinal fluid (124 mM NaCl, 2 mM KCl, 2 mM MgSO_4_, 2 mM CaCl_2_, 1.25 mM KH_2_PO_4_, 26 mM NaHCO_3_, 10 mM glucose) was inserted into the optic tectum (MultiClamp 700B amplifier, Digidata 1550 digitizer, Axon Instruments, Burlingame, CA, USA). The recordings for each larva were performed within 20 min. Clampfit 10.2 software (Molecular Devices Corporation, San Jose, CA, USA) and custom-written R script for Windows were used for analysis purposes.

### 4.10. HPLC-ESI-QTOF-MS/MS Analysis of Neurotransmitters Levels in Zebrafish

Larvae (6 dpf, *n* = 5–9 samples/group) were exposed to 37.5 µM 6-GIN or Veh for 24 h with subsequent 90 min long exposure to 20 mM PTZ. Next, whole zebrafish larvae were collected in a tube at *n* = 100/sample. When the excess fluid was removed, the larvae were snap-frozen in liquid nitrogen before adding 200 µL of cold milli-Q water and acetonitrile (stored at −20 °C to each tube). The samples were sonicated at 4 °C and the supernatant collected after centrifuging for five minutes at 24 000 rpm.

Quantitative analysis of GLU and GABA in the zebrafish samples was performed using the same chromatograph coupled with a mass spectrometer, as described above. Additionally, the levels of 5-HT and DA were determined using the same methodology as described for GLU and GABA to give more comprehensive information on the influence of 6-GIN on zebrafish larvae (see Figures S1A,B and S2 in the Supplementary Materials file). For the analysis of neurotransmitters’ level in zebrafish, the obtained zebrafish supernatants were filtered through nylon syringe filters (pore diameter of 0.22 µm (Sigma Aldrich, St. Louis, MO, USA) and subjected to the HPLC-MS analysis using a ReproSil-Pur 120 Si column (150 × 2.0 mm, 5 µm) by Dr. Maisch (Ammerbuch, Germany). The following gradient of acetonitrile with 0.1% formic acid (solvent B) in 0.1% aqueous solution of formic acid was applied: 0 min 98% B, 5 min 80% B, 40 min 77.5% B, 45 min 30% B, and 50 min 98% B.

The analysis time was set at 70 min, the flow rate at 0.2 mL/min, the temperature of column oven at 25 °C, and the injection volume at 10 µL. The mass spectra were recorded within the *m*/*z* range of 50–1200 in the following mass spectrometer settings: gas and sheath gas temperatures of 275 and 300 °C, gas flow rates of 12 L/min, nebulizer pressure of 35 psig, capillary voltage of 2750 V, fragmentor voltage of 80 V, collision energies of 10 and 20 V, and skimmer voltage of 65 V. The method was data-dependent and provided MS/MS spectra for two of the most intensive signals in a given scan; however, after the collection of 1 spectrum, the fragmented signals were excluded for the following 0.3 min from the following fragmentations.

The quantitative analysis was performed based on the calibration curves that were prepared for the corresponding reference compounds by preparing 5 different concentrations (corresponding to those present in the analyzed samples) of every reference compound and by injecting them in the method described above. The calibration curve equations obtained for GABA and GLU were as follows: y = 1,655,578x + 5,156,982 (R^2^ = 0.9966) and y = 12,902,625x − 179,259 (R^2^ = 0.9944), respectively.

### 4.11. qRT-PCR Assessment

Larvae (7 dpf, *n* = 3 samples/group) were pre-treated with 6-GIN (37.5 µM) or Veh for 24 h with 90 min long follow up exposure to 20 mM PTZ. Next, zebrafish larvae were collected in a pool of *n* = 10/sample. RNA was extracted using TRIZOL and cDNA synthesized using SuperScript™ IV First-Strand Synthesis System (Invitrogen). cDNA was amplified using PowerUp™ SYBR™ Green Master Mix (Applied Biosystems) according to the manufacturer’s instructions. Relative enrichment was computed according to the 2^−ΔΔt^ method, while expression levels were normalized against the housekeeping gene glyceraldehyde 3-phosphate dehydrogenase (*gapdh*) [72].

Primer sequences:

gapdh_f_5′GTGGAGTCTACTGGTGTCTTC3′

gapdh_r_5′GTGCAGGAGGCATTGCTTACA3′

gabra1_f_5′GTAGCCTATGCCACAGCCAT3′

gabra1_r_5′TCTTTTGCTTTTCTGGCACCAC3′

grin1a_f_ 5′CGAGCCCAAGATTGTGAACA3′

grin1a_r_5′CCTGGGTGACGGCATCTTTA3′

grin2b_f_5′AGTCGGGTAAATTGGATGCATT3′

grin2b_r_5′CCAGCTTACAGCCCTCATCAC3′

gria1a_f_5′AAGTTCCCAAGCGAAGACTGAA3′

gria1a_r_5′GTTCTGGAAAGCTGTGGACATG3′

gria2a_f_5′GGAGACTGCCTGGCCAATC3′

gria2a_r_5′TCCACACGCACCTGTTTAAGAG3′

gria3b_f_5′GCAGGTTGTGACACTTGGAAAG3′

gria3b_r_5′AGGAAGACCTTGTCCAGGCTAA3′

### 4.12. Molecular Docking of 6-GIN to GluN2B-Containing NMDA Receptor

The available crystal structure of the GluN1/GluN2B NMDA receptor at 3.59 Å atomic resolution (PDB ID: 4TLL) [73] was used to perform the molecular docking. For the molecular docking procedure, the 6-GIN structure was taken from PubChem Database (PubChem CID 442793) and geometrically optimized using the semi-empirical AM1 method found in Spartan 10V.1.1.0 (Wavefunction, Inc., Irvine, CA, USA), as previously described [74]. AutoDock Vina [75] was used for docking simulations of flexible ligand into the ATDs, LBDs, and TMD of the rigid GluN1/GluN2B NMDA model. The chosen dimensions for the grid maps were 36 Å × 36 Å × 36 Å for the ATD, 22 Å × 22 Å × 22 Å for the LBD (glutamate-binding site), and 36 Å × 36 Å × 36 Å for TMD, which covered the ion channel pore. The parameters used with AutoDock Vina were exhaustiveness (570) and the number of ligand poses (20). The energetically lower poses were selected from each cluster of superposed poses of 6-GIN at each binding sites (i.e, ATD, LBD, and TMD) and presented in Table 1.

Moreover, to determine whether the residues involved in 6-GIN binding to the GluN2B-containing NMDA receptor are identical in human and zebrafish, the sequence alignment of the GluN2B subunit was generated using Multiple Sequence Alignment Muscle (https://www.ebi.ac.uk/Tools/msa/muscle/, accessed on 15 May 2021). The amino acid sequences of the GluN2B subunit were obtained from the UniProt database implemented in ExPASy Bioinformatics Resource Portal [76].

### 4.13. Statistical Analysis

The results are presented as mean ± SEM. The data were analyzed using one-way or two-way ANOVA with repeated measures with Tukey’s or Bonferroni’s *post hoc* tests, respectively. Student’s *t*-test was used when required. The criterion for statistical significance was set at *p* < 0.05. For statistical purposes and figure generation, GraphPad Prism 8.3.0 version (San Diego, CA, USA) was applied.

## Figures and Tables

**Table 1:** Molecular interactions of 6-GIN with GluN1/GluN2B NMDA receptor.

Binding Site Location	Docking Results	
	Ligand Poses	Residues Involved in 6-GIN Binding
ATD	Orientation 1	**GluN1:** Ala75, Ala102, Pro106, Ser108, Tyr109, Thr110, Gly112, Phe113, Arg115, Lys131, Ser132, Ile133, Leu135 **GluN2B:** Pro73, Ile77, Ala102, Gln105, Ile106, Phe109, Phe171, Pro172
	Orientation 2	**GluN1:** Ala71, His101, Leu102, Thr103, Thr105, Pro106, Tyr109, Ile133 **GluN2B:** Gln105, Asp108, Phe109, Val112, Gln113, Met129, Ala130, Glu133, Ser136
Glutamate-binding site (LBD)	Orientation 1	**GluN1:** Lys529, Pro530, Ser763, Ile766, Leu767, His770, Glu771 **GluN2B:** Thr507, Ile508, Asn509, Pro521, Thr525, Gly526, Ile527, Lys529, Pro530, Ser673, Thr674, Asn677, Asp715, Ala716, Phe740, Ala741, Thr742, Thr743, Gly744, Ile766, Leu767, His770
	Orientation 2	**GluN2B:** Leu404, Glu405, Glu406, Lys478, His479, Ser505, Leu506, Thr507, Glu675, Val669, Gly672, Ser673, Thr674, Val697, Tyr714, Asp715, Val718, Tyr745
TMD		**GluN1 (1):** Met631, Val634, Ala635, Thr638 **GluN1 (2):** Thr630, Met631, Val634, Thr638, **GluN2B (1):** Asn598, Asn599, Ala622, Val623, Leu626, Ala627, Thr630 **GluN2B (2):** Asn598, Val623, Leu626, Ala627, Thr630

## Data Availability

The raw data supporting the results are presented in the manuscript and in the Supplementary File.

## Supplementary Materials

The following are available online at https://www.mdpi.com/article/10.3390/ijms22147745/s1, Figure S1: Determination of (A) serotonin, and (B) dopamine in 7 days old zebrafish larvae by HPLC-MS., Figure S2: The total ion chromatogram (A) of the water:acetonitrile extract from zebrafish larvae with extracted ion chromatograms of serotonin and dopamine in 7-day-old zebrafish larvae by HPLC-MS.

## References

[1] Moshé S.L., Perucca E., Ryvlin P., Tomson T. (2015). Epilepsy: New advances. Lancet.

[2] Chen T., Giri M., Xia Z., Subedi Y.N., Li Y. (2017). Genetic and epigenetic mechanisms of epilepsy: A review. Neuropsychiatr. Dis. Treat..

[3] Myers K.A., Johnstone D.L., Dyment D.A. (2019). Epilepsy genetics: Current knowledge, applications, and future directions. Clin. Genet..

[4] Perucca P., Bahlo M., Berkovic S.F. (2020). The Genetics of Epilepsy. Annu. Rev. Genom. Hum. Genet..

[5] Perucca P., Gilliam F.G. (2012). Adverse effects of antiepileptic drugs. Lancet Neurol..

[6] Löscher W., Potschka H., Sisodiya S.M., Vezzani A. (2020). Drug Resistance in Epilepsy: Clinical Impact, Potential Mechanisms, and New Innovative Treatment Options. Pharmacol. Rev..

[7] Gawel K., Langlois M., Martins T., van der Ent W., Tiraboschi E., Jacmin M., Crawford A.D., Esguerra C.V. (2020). Seizing the moment: Zebrafish epilepsy models. Neurosci. Biobehav. Rev..

[8] Yaksi E., Jamali A., Diaz Verdugo C., Jurisch-Yaksi N. (2021). Past, present and future of zebrafish in epilepsy research. FEBS J..

[9] Howe K., Clark M.D., Torroja C.F., Torrance J., Berthelot C., Muffato M., Collins J.E., Humphray S., McLaren K., Matthews L. (2013). The zebrafish reference genome sequence and its relationship to the human genome. Nature.

[10] Afrikanova T., Serruys A.S., Buenafe O.E., Clinckers R., Smolders I., de Witte P.A., Crawford A.D., Esguerra C.V. (2013). Validation of the zebrafish pentylenetetrazol seizure model: Locomotor versus electrographic responses to antiepileptic drugs. PLoS ONE.

[11] Leclercq K., Afrikanova T., Langlois M., de Prins A., Buenafe O.E., Rospo C.C., van Eeckhaut A., de Witte P.A., Crawford A.D., Smolders I. (2015). Cross-species pharmacological characterization of the allylglycine seizure model in mice and larval zebrafish. Epilepsy Behav..

[12] Zhang Y., Vanmeert M., Siekierska A., Ny A., John J., Callewaert G., Lescrinier E., Dehaen W., de Witte P.A.M., Kaminski R.M. (2017). Inhibition of glutamate decarboxylase (GAD) by ethyl ketopentenoate (EKP) induces treatment-resistant epileptic seizures in zebrafish. Sci. Rep..

[13] Gawel K., Kukula-Koch W., Nieoczym D., Stepnik K., Ent W.V., Banono N.S., Tarabasz D., Turski W.A., Esguerra C.V. (2020). The Influence of Palmatine Isolated from Berberis sibirica Radix on Pentylenetetrazole-Induced Seizures in Zebrafish. Cells.

[14] Nieoczym D., Socała K., Gawel K., Esguerra C.V., Wyska E., Wlaź P. (2019). Anticonvulsant Activity of Pterostilbene in Zebrafish and Mouse Acute Seizure Tests. Neurochem. Res..

[15] Gong G., Chen H., Kam H., Chan G., Tang Y.X., Wu M., Tan H., Tse Y.C., Xu H.X., Lee S.M. (2020). In Vivo Screening of Xanthones from Garcinia oligantha Identified Oliganthin H as a Novel Natural Inhibitor of Convulsions. J. Nat. Prod..

[16] Aourz N., Serruys A.-S.K., Chabwine J.N., Balegamire P.B., Afrikanova T., Edrada-Ebel R., Grey A.I., Kamuhabwa A.R., Walrave L., Esguerra C.V. (2019). Identification of GSK-3 as a Potential Therapeutic Entry Point for Epilepsy. ACS Chem. Neurosci..

[17] Orellana-Paucar A.M., Serruys A.S., Afrikanova T., Maes J., De Borggraeve W., Alen J., León-Tamariz F., Wilches-Arizábala I.M., Crawford A.D., de Witte P.A. (2012). Anticonvulsant activity of bisabolene sesquiterpenoids of Curcuma longa in zebrafish and mouse seizure models. Epilepsy Behav..

[18] Kukula-Koch W., Czernicka L., Xiao J., Sarker S.D., Asakawa Y. (2019). Gingerols and Shogaols from Food. Handbook of Dietary Phytochemicals.

[19] Simon A., Darcsi A., Kery A., Riethmuller E. (2020). Blood-brain barrier permeability study of ginger constituents. J. Pharm. Biomed. Anal..

[20] Kukula-Koch W., Koch W., Czernicka L., Głowniak K., Asakawa Y., Umeyama A., Marzec Z., Kuzuhara T. (2018). MAO-A Inhibitory Potential of Terpene Constituents from Ginger Rhizomes—A Bioactivity Guided Fractionation. Molecules.

[21] Banerjee S., Banerjee J., Mullick H., Ghosh A. (2011). *Zingiber officinale*: A natural gold. Int. J. Pharma Biol. Sci..

[22] Han J.J., Li X., Ye Z.Q., Lu X.Y., Yang T., Tian J., Wang Y.Q., Zhu L., Wang Z.Z., Zhang Y. (2019). Treatment with 6-Gingerol Regulates Dendritic Cell Activity and Ameliorates the Severity of Experimental Autoimmune Encephalomyelitis. Mol. Nutr. Food Res..

[23] Ha S.K., Moon E., Ju M.S., Kim D.H., Ryu J.H., Oh M.S., Kim S.Y. (2012). 6-Shogaol, a ginger product, modulates neuroinflammation: A new approach to neuroprotection. Neuropharmacology.

[24] Hosseini A., Mirazi N. (2014). Acute administration of ginger (*Zingiber officinale* rhizomes) extract on timed intravenous pentylenetetrazol infusion seizure model in mice. Epilepsy Res..

[25] Hosseini A., Mirazi N. (2015). Alteration of pentylenetetrazole-induced seizure threshold by chronic administration of ginger (*Zingiber officinale*) extract in male mice. Pharm. Biol..

[26] Brillatz T., Kubo M., Takahashi S., Jozukuri N., Takechi K., Queiroz E.F., Marcourt L., Allard P.M., Fish R., Harada K. (2020). Metabolite Profiling of Javanese Ginger *Zingiber purpureum* and Identification of Antiseizure Metabolites via a Low-Cost Open-Source Zebrafish Bioassay-Guided Isolation. J. Agric. Food Chem..

[27] Murugesan A., Rani M.R.S., Hampson J., Zonjy B., Lacuey N., Faingold C.L., Friedman D., Devinsky O., Sainju R.K., Schuele S. (2018). Serum serotonin levels in patients with epileptic seizures. Epilepsia.

[28] Gilliam F.G., Hecimovic H., Gentry M.S. (2021). Serotonergic therapy in epilepsy. Curr. Opin. Neurol..

[29] Akyuz E., Polat A.K., Eroglu E., Kullu I., Angelopoulou E., Paudel Y.N. (2021). Revisiting the role of neurotransmitters in epilepsy: An updated review. Life Sci..

[30] Bozzi Y., Borrelli E. (2013). The role of dopamine signaling in epileptogenesis. Front. Cell. Neurosci..

[31] Gauthier M.L., Douat J., Vachon P., Beaudry F. (2011). Characterization of [6]-gingerol metabolism in rat by liquid chromatography electrospray tandem mass spectrometry. Biomed. Chromatogr..

[32] Monesson-Olson B., McClain J.J., Case A.E., Dorman H.E., Turkewitz D.R., Steiner A.B., Downes G.B. (2018). Expression of the eight GABAA receptor α subunits in the developing zebrafish central nervous system. PLoS ONE.

[33] Samarut É., Swaminathan A., Riché R., Liao M., Hassan-Abdi R., Renault S., Allard M., Dufour L., Cossette P., Soussi-Yanicostas N. (2018). γ-Aminobutyric acid receptor alpha 1 subunit loss of function causes genetic generalized epilepsy by impairing inhibitory network neurodevelopment. Epilepsia.

[34] Cossette P., Liu L., Brisebois K., Dong H., Lortie A., Vanasse M., Saint-Hilaire J.M., Carmant L., Verner A., Lu W.Y. (2002). Mutation of GABRA1 in an autosomal dominant form of juvenile myoclonic epilepsy. Nat. Genet..

[35] Carvill G.L., Weckhuysen S., McMahon J.M., Hartmann C., Møller R.S., Hjalgrim H., Cook J., Geraghty E., O’Roak B.J., Petrou S. (2014). GABRA1 and STXBP1: Novel genetic causes of Dravet syndrome. Neurology.

[36] Hunt R.F., Hortopan G.A., Gillespie A., Baraban S.C. (2012). A novel zebrafish model of hyperthermia-induced seizures reveals a role for TRPV4 channels and NMDA-type glutamate receptors. Exp. Neurol..

[37] Maljevic S., Reid C.A., Petrou S. (2017). Models for discovery of targeted therapy in genetic epileptic encephalopathies. J. Neurochem..

[38] Hanada T. (2020). Ionotropic Glutamate Receptors in Epilepsy: A Review Focusing on AMPA and NMDA Receptors. Biomolecules.

[39] Hwang J., Kim H.-S., Seok J.-W., Kim J.-D., Koun S., Park S.-Y., Lee J., Kim H.S., Kim H.-S., Kim K.S. (2009). Transcriptome analysis of the zebrafish mind bomb mutant. Mol. Genet. Genom..

[40] Cox J.A., Kucenas S., Voigt M.M. (2005). Molecular characterization and embryonic expression of the family of N-methyl-D-aspartate receptor subunit genes in the zebrafish. Dev. Dyn..

[41] Wasilewska I., Gupta R.K., Palchevska O., Kuźnicki J. (2019). Identification of Zebrafish Calcium Toolkit Genes and Their Expression in the Brain. Genes.

[42] Klee E.W., Schneider H., Clark K.J., Cousin M.A., Ebbert J.O., Hooten W.M., Karpyak V.M., Warner D.O., Ekker S.C. (2012). Zebrafish: A model for the study of addiction genetics. Hum. Genet..

[43] Hoppmann V., Wu J.J., Søviknes A.M., Helvik J.V., Becker T.S. (2008). Expression of the eight AMPA receptor subunit genes in the developing central nervous system and sensory organs of zebrafish. Dev. Dyn..

[44] Koch W., Kukula-Koch W., Marzec Z., Kasperek E., Wyszogrodzka-Koma L., Szwerc W., Asakawa Y. (2017). Application of Chromatographic and Spectroscopic Methods towards the Quality Assessment of Ginger (*Zingiber officinale*) Rhizomes from Ecological Plantations. Int. J. Mol. Sci..

[45] Chung W.Y., Jung Y.J., Surh Y.J., Lee S.S., Park K.K. (2001). Antioxidative and antitumor promoting effects of [6]-paradol and its homologs. Mutat. Res..

[46] Tang S., Song H., Liu E., Qi J. (2014). Isolation and Purification of Gingerols from Ginger by High-Speed Counter-Current Chromatography. Asian J. Chem..

[47] Qiao Q., Du Q. (2011). Preparation of the monomers of gingerols and 6-shogaol by flash high speed counter-current chromatography. J. Chromatogr. A.

[48] Lee C., Park G.H., Kim C.Y., Jang J.H. (2011). [6]-Gingerol attenuates beta-amyloid-induced oxidative cell death via fortifying cellular antioxidant defense system. Food Chem. Toxicol..

[49] Kim C.Y., Seo Y., Lee C., Park G.H., Jang J.H. (2018). Neuroprotective Effect and Molecular Mechanism of [6]-Gingerol against Scopolamine-Induced Amnesia in C57BL/6 Mice. Evid. Based Complement. Altern. Med..

[50] Kabuto H., Nishizawa M., Tada M., Higashio C., Shishibori T., Kohno M. (2005). Zingerone [4-(4-hydroxy-3-methoxyphenyl)-2-butanone] prevents 6-hydroxydopamine-induced dopamine depression in mouse striatum and increases superoxide scavenging activity in serum. Neurochem. Res..

[51] Waggas A.M. (2009). Neuroprotective evaluation of extract of ginger (*Zingiber officinale*) root in monosodium glutamate-induced toxicity in different brain areas male albino rats. Pak. J. Biol. Sci..

[52] Deng X.Y., Xue J.S., Li H.Y., Ma Z.Q., Fu Q., Qu R., Ma S.P. (2015). Geraniol produces antidepressant-like effects in a chronic unpredictable mild stress mice model. Physiol. Behav..

[53] Martinez D.M., Barcellos A., Casaril A.M., Savegnago L., Lernardão E.J. (2014). Antidepressant-like activity of dehydrozingerone: Involvement of the serotonergic and noradrenergic systems. Pharmacol. Biochem. Behav..

[54] Moon S., Lee M.S., Jung S., Kang B., Kim S.Y., Park S., Son H.Y., Kim C.T., Jo Y.H., Kim I.H. (2017). High Hydrostatic Pressure Extract of Ginger Exerts Antistress Effects in Immobilization-Stressed Rats. J. Med. Food.

[55] Vishwakarma S.L., Pal S.C., Kasture V.S., Kasture S.B. (2002). Anxiolytic and antiemetic activity of *Zingiber officinale*. Phytother. Res..

[56] Sharma P.K., Singh V., Ali M., Kumar S. (2016). Effect of ethanolic extract of *Zingiber officinale* Roscoe on central nervous system activity in mice. Indian J. Exp. Biol..

[57] Reyes-Carmona J., Yousef G.G., Martínez-Peniche R.A., Lila M.A. (2005). Antioxidant Capacity of Fruit Extracts of Blackberry (Rubus sp.) Produced in Different Climatic Regions. J. Food Sci..

[58] Teixeira A., Eiras-Dias J., Castellarin S.D., Gerós H. (2013). Berry phenolics of grapevine under challenging environments. Int. J. Mol. Sci..

[59] Jung M.Y., Lee M.K., Park H.J., Oh E.-B., Shin J.Y., Park J.S., Jung S.Y., Oh J.-H., Choi D.-S. (2017). Heat-induced conversion of gingerols to shogaols in ginger as affected by heat type (dry or moist heat), sample type (fresh or dried), temperature and time. Food Sci. Biotechnol..

[60] Hosseini A., Mirazi N., Gomar A. (2016). Protective effect of ginger against the pentylenetetrazole-induced seizure threshold model in streptozocin treated-diabetic mice. Physiol. Pharmacol..

[61] Ramanjaneyulu R., Ticku M.K. (1984). Interactions of pentamethylenetetrazole and tetrazole analogues with the picrotoxinin site of the benzodiazepine-GABA receptor-ionophore complex. Eur. J. Pharmacol..

[62] Kalueff A.V. (2007). Mapping convulsants’ binding to the GABA-A receptor chloride ionophore: A proposed model for channel binding sites. Neurochem. Int..

[63] Bandara S.B., Carty D.R., Singh V., Harvey D.J., Vasylieva N., Pressly B., Wulff H., Lein P.J. (2020). Susceptibility of larval zebrafish to the seizurogenic activity of GABA type A receptor antagonists. Neurotoxicology.

[64] Moradi-Afrapoli F., Ebrahimi S.N., Smiesko M., Hamburger M. (2017). HPLC-Based Activity Profiling for GABA(A) Receptor Modulators in Extracts: Validation of an Approach Utilizing a Larval Zebrafish Locomotor Assay. J. Nat. Prod..

[65] Buszewski B., Noga S. (2012). Hydrophilic interaction liquid chromatography (HILIC)—A powerful separation technique. Anal. Bioanal. Chem..

[66] Wang L.S., Zhang M.D., Tao X., Zhou Y.F., Liu X.M., Pan R.L., Liao Y.H., Chang Q. (2019). LC-MS/MS-based quantification of tryptophan metabolites and neurotransmitters in the serum and brain of mice. J. Chromatogr. B Analyt. Technol. Biomed. Life Sci..

[67] Blanco M.E., Mayo O.B., Bandiera T., de Pietri Tonelli D., Armirotti A. (2020). LC–MS/MS analysis of twelve neurotransmitters and amino acids in mouse cerebrospinal fluid. J. Neurosci. Methods.

[68] Hussein U.K., Hassan N.E.Y., Elhalwagy M.E.A., Zaki A.R., Abubakr H.O., Nagulapalli Venkata K.C., Jang K.Y., Bishayee A. (2017). Ginger and Propolis Exert Neuroprotective Effects against Monosodium Glutamate-Induced Neurotoxicity in Rats. Molecules.

[69] Hegazy H., Ali E. (2011). Modulation of monoamines and amino-acids neurotransmitters in cerebral cortex and hippocampus of female senile rats by ginger and lipoic acid. Afr. J. Pharm. Pharmacol..

[70] Rogawski M.A. (2011). Revisiting AMPA receptors as an antiepileptic drug target. Epilepsy Curr..

[71] Tiraboschi E., Martina S., van der Ent W., Grzyb K., Gawel K., Cordero-Maldonado M.L., Poovathingal S.K., Heintz S., Satheesh S.V., Brattespe J. (2020). New insights into the early mechanisms of epileptogenesis in a zebrafish model of Dravet syndrome. Epilepsia.

[72] McCurley A.T., Callard G.V. (2008). Characterization of housekeeping genes in zebrafish: Male-female differences and effects of tissue type, developmental stage and chemical treatment. BMC Mol. Biol..

[73] Lee C.H., Lü W., Michel J.C., Goehring A., Du J., Song X., Gouaux E. (2014). NMDA receptor structures reveal subunit arrangement and pore architecture. Nature.

[74] Targowska-Duda K.M., Kaczor A.A., Jozwiak K., Arias H.R. (2019). Molecular interactions of type I and type II positive allosteric modulators with the human α7 nicotinic acetylcholine receptor: An in silico study. J. Biomol. Struct. Dyn..

[75] Trott O., Olson A.J. (2010). AutoDock Vina: Improving the speed and accuracy of docking with a new scoring function, efficient optimization, and multithreading. J. Comput. Chem..

[76] Gasteiger E., Gattiker A., Hoogland C., Ivanyi I., Appel R.D., Bairoch A. (2003). ExPASy: The proteomics server for in-depth protein knowledge and analysis. Nucleic Acids Res..

