# Supplementary material for: 6-Gingerol, a Major Constituent of Zingiber officinale Rhizoma, Exerts Anticonvulsant Activity in the Pentylenetetrazole-Induced Seizure Model in Larval Zebrafish
Source: Int J Mol Sci. 2021 Jul 20;22(14):7745. doi: 10.3390/ijms22147745 (PMC8305044; doi:10.3390/ijms22147745)

SUPPLEMENTARY FILE

6-Gingerol, a Major Constituent of *Zingiber officinale* Rhizoma and Exerts Anticonvulsant Activity in the Pentylenetetrazole-Induced Seizure Model in Larval Zebrafish

Kinga Gawel [1, 2,*], Wirginia Kukula-Koch [3], Nancy Saana Banono [1,†], Dorota Nieoczym [4,†], Katarzyna Targowska-Duda [5], Lidia Czernicka [6], Jolanta Parada-Turska [7] and Camila V. Esguerra [1]

Figure S1. Determination of A) serotonin, and B) dopamine in 7 days old zebrafish larvae by HPLC-MS. After 24-h incubation in 6-GIN (37.5 μM), zebrafish larvae were exposed to acute dose of PTZ (20 mM) for 90 min. Next, whole zebrafish larvae were collected in a pool of n=100/sample. Data were analyzed using one-way ANOVA followed by the Tukey's *post-hoc* test. Data are depicted as mean ± SEM. Veh + veh (*n* = 9), 6-GIN 37.5 + Veh (*n* = 5), Veh + PTZ (*n* = 5), 6-GIN 37.5 + PTZ (*n* = 5). [&&&] P<0.001, [&&] P<0.01, vs. Veh + PTZ. 5-HT- serotonin, 6-GIN – 6-gingerol, DA- dopamine, PTZ – pentylenetetrazole, Veh – vehicle. n- refers to total number of larvae.

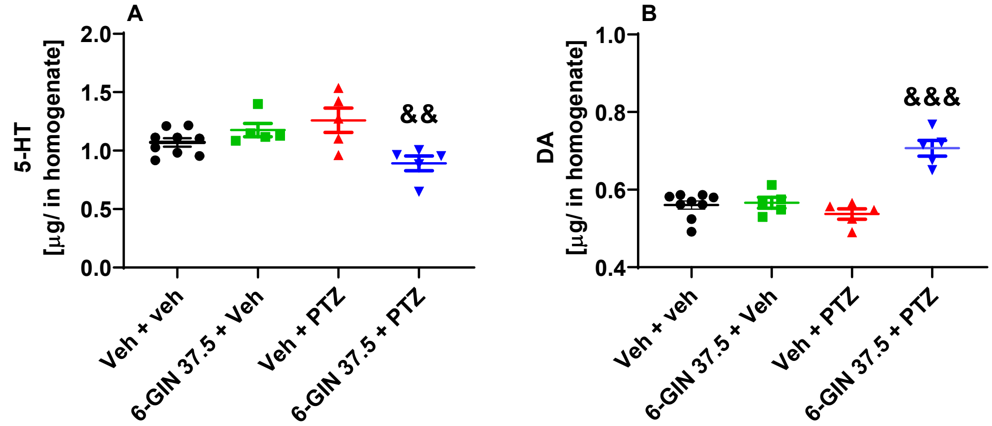

Figure S2. The total ion chromatogram (A) of the water:acetonitrile extract from zebrafish larvae with extracted ion chromatograms of serotonin and dopamine in 7 days old zebrafish larvae by HPLC-MS.

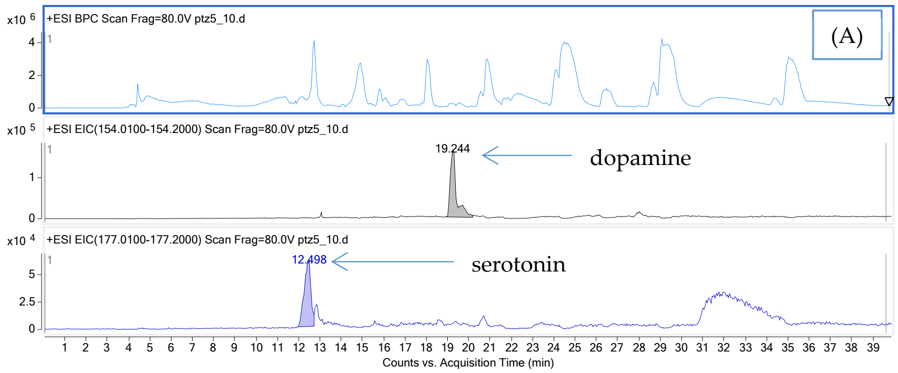

Supplement: Supplementary file 1 [file ijms-22-07745-s001.zip › ijms-1251526-supplementary.pdf]
